# 20(S)-Ginsenoside Rg3 Protects Kidney from Diabetic Kidney Disease via Renal Inflammation Depression in Diabetic Rats

**DOI:** 10.1155/2020/7152176

**Published:** 2020-03-18

**Authors:** Tong Zhou, Lin Sun, Shuo Yang, You Lv, Yue Cao, Xiaokun Gang, Guixia Wang

**Affiliations:** ^1^Department of Endocrinology and Metabolism, The First Hospital of Jilin University, 130061 Changchun, Jilin Province, China; ^2^Key Laboratory of Organ Regeneration & Transplantation of the Ministry of Education, The First Hospital of Jilin University, 130061 Changchun, Jilin Province, China

## Abstract

20(S)-Ginsenoside Rg3 (20(S)-Rg3) has been shown to induce apoptosis by interfering with several signaling pathways. Furthermore, it has been reported to have anticancer and antidiabetic effects. In order to detect the protective effect of 20(S)-Rg3 on diabetic kidney disease (DKD), diabetic rat models which were established by administering high-sugar, high-fat diet combined with intraperitoneal injection of streptozotocin (STZ), and age-matched wild-type (WT) rat were given 20(S)-Rg3 for 12 weeks, with three groups: control group (normal adult rats with saline), diabetic group (diabetic rats with saline), and 20(S)-Rg3 treatment group (diabetic rats with 20(S)-Rg3 (10 mg/kg body weight/day)). The biochemical indicators and the changes in glomerular basement membrane and mesangial matrix were detected. TUNEL staining was used to detect glomerular and renal tubular cell apoptosis. Immunohistochemical staining was used to detect the expression of fibrosis factors and inflammation factors in rat kidney tissues. Through periodic acid-Schiff staining, we observed that the change in renal histology was improved and renal tubular epithelial cell apoptosis decreased significantly by treatment with 20(S)-Rg3. Plus, the urine protein decreased in the rats with the 20(S)-Rg3 treatment. Fasting blood glucose, creatinine, total cholesterol, and triglyceride levels in the 20(S)-Rg3 treatment group were all lower than those in the diabetic group. Mechanistically, 20(S)-Rg3 dramatically downregulated the expression of TGF-*β*1, NF-*κ*B65, and TNF-*α* in the kidney. These resulted in a significant prevention of renal damage from the inflammation. The results of the current study suggest that 20(S)-Rg3 could potentially be used as a novel treatment against DKD.

## 1. Introduction

Diabetic kidney disease (DKD) refers to glomerular sclerosis caused by diabetic microvascular lesions and is also known as diabetic glomerular sclerosis. DKD is one of the most common chronic complications of diabetes mellitus (DM), and it may eventually cause end-stage kidney disease [1]. The clinical manifestations of DKD include hypertension, proteinuria, edema, and renal insufficiency. DKD mortality for patients with diabetes is more than 60%, and it is the main cause of death in patients with diabetes.

The pathogenesis of DKD is not yet clear. Recent studies have shown that inflammation is crucial in the initiation and development of DKD [2, 3]. Adhesion molecules, inflammatory chemokines, and inflammatory markers play important roles in the development of DKD [4]. DM may lead to the production and release of various inflammatory markers, such as transforming growth factor beta 1 (TGF-*β*1), connective tissue growth factor (CTGF), platelet-derived growth factor (PDGF), and nuclear transcription factors-kappa B (NF-*κ*B), predominantly, vascular endothelial cell growth factor (VEGF) and tumor necrosis factor-*α* (TNF-*α*) [5, 6]. These factors interact with and restrict each other, forming a complex network of cytokines to promote the occurrence and development of DKD. Cytokines play a critical role in the accumulation of extracellular matrix (ECM), which is the pathological basis of DKD [7]. There is increased expression of renal cytokines, such as TGF-*β*1, in DKD, and this abnormality could further reduce ECM degradation and enhance ECM synthesis, which could lead to fibrosis of glomerulus and renal tubules.

Traditional treatment for DKD includes controlling blood glucose and blood pressure, low-protein diet, and taking drugs for lipid lowering and interference of renin-angiotensin system [8]. Although these treatments can delay the development of DKD, the incidence and mortality of DKD remain high; therefore, there is an urgent need to develop drugs that can effectively prevent DKD occurrence and development.

Ginsenoside is a major pharmacological active ingredient of ginseng; however, ginseng exerts its activity mainly via its purification into the monomer saponin [9, 10]. To date, more than 40 kinds of ginseng saponin monomers have been isolated from ginseng, among which, Rg1, Rg3, Rb1, Rd, Re, and Rh1 are the mostly studied [11]. Ginsenoside Rg3 monomer is the monomer saponin extracted from red ginseng. There are two isomers, 20(R)-ginsenoside Rg3 (20(R)-Rg3) and 20(S)-ginsenoside Rg3 (20(S)-Rg3). Studies on 20(R)-Rg3 and 20(S)-Rg3 have found that although they have the same pharmacological effects, 20(S)-Rg3 has higher purity and better solubility in water than 20(R)-Rg3 [10]. Recently, it is shown that 20(S)-Rg3 exerted liver protection against hepatotoxicity by reducing the levels of oxidative stress and inflammatory response in liver [12]. It was also proved to attenuate acute lung injury by decreasing the levels of proinflammatory cytokines [13]. 20(S)-Rg3 interferes with the occurrence and development of diabetic retinopathy by downregulating the expression of TNF-*α* and VEGF in diabetic rat retinal tissues [14]. The intramuscular injection of 20(S)-Rg3 has more effects in vivo and in vitro (animal and phase I clinical research), higher bioavailability, and good continuous dosing security. Moreover, 20(S)-Rg3 is more suitable for application in clinical medicine, such as a novel inhibitor of autophagy [15] or protecting INS-1 cell death from intermittent high glucose [16]. It also has been confirmed that 20(S)-Rg3 is a compound that has many pharmacological effects such as anticancer [17], inhibition of brain injury [18], and diabetic kidney injury resistance [19]. Thus, 20(S)-Rg3 therapy has now become a nontraditional therapy for diabetes and its complications treatment. However, there is less evidence to illustrate the mechanism about the protection effect of 20(S)-Rg3 on diabetic kidney disease.

In this study, we aimed to investigate the protective effects of 20(S)-Rg3 on DKD by measuring the expression of cytokines and various inflammatory markers and to determine the relationship with inflammation process. The findings of this study provide a theoretical basis for the nontraditional clinical treatment of DKD and for delaying the process of DKD.

## 2. Materials and Methods

### 2.1. Experimental Animals and Reagents

Thirty 4-week-old male Wistar rats (190 ± 20 g) were purchased from the Animal Experiment Center at Basic Medical College of Jilin University (Animal Certificate Number: SCXK-(JI)2015-0001). 20(S)-Rg3 monomer was bought from the pharmacy of Jilin University (Purity 99%). Streptozotocin (STZ) was purchased from Sigma Chemical Co., St. Louis, MO, USA. Total cholesterol (TC) (A111-1), triglycerides (TG) (A110-1), creatinine (Cr) (C011-2), and BCA protein assay kits (A045-4) were purchased from Nanjing Jiancheng Institute of Biological Engineering, China. TGF-*β*1 (bs-0103R), CTGF (bs-0743R), NF-*κ*Bp65 (bs-0465R), and TNF-*α* (bs-0078R) antibodies were purchased from Beijing Bioss Company, China. Surfactant protein (SP) allergic kit (KIT-9710), endogenous biotin blocking kit (BLK-0001), and diaminobenzidine (DAB) chromogenic agent (DAB-0031) were purchased from Fuzhou Maxim Company, China. TUNEL kit (11684817910) was purchased from Roche, Germany.

### 2.2. Cell Culture and Treatment

Rat renal tubular epithelial cells NRK-52E were donated by Professor Xin Ying from the Basic Medical College of Jilin University. NRK-52E cells were cultured in DMEM (Gibco, SUA) medium containing 10% FBS (Gibco, SUA), routinely cultured at 37°C, 5% CO_2_ incubator (Panasonic, Japan), and subcultured once every 48 h. Take the cells in logarithmic growth phase and digest with 0.25% trypsin (Sigma, USA) to make a single cell suspension. The cells are seeded in a 96-well culture plate, 2000 cells/well, cultured for 24 hours, and the original culture solution is aspirated. Different concentration of palmitic acid (0, 50, 100, and 150 *μ*M) was added, the culture time was 24 h, at least 3 in each group in parallel. Observe the morphology and growth of the cells under a microscope (Olympus, Japan).

### 2.3. Western Blot Detection of Inflammation-Related Proteins

Take the cells in logarithmic phase, adjust the cell density to 1 × 10^5^/mL, inoculate 10 × Petri dishes, 30 × 10^4^ cells per well, culture for 24 h, then add different concentrations of palmitic acid (50, 100, and 150 *μ*M), 3 parallel in each group. After 24 hours of incubation, add different concentrations of 20(S)-Rg3 (25 *μ*M and 50 *μ*M). After 24 hours of incubation, discard the original culture solution and wash it twice with PBS. Add 200 *μ*L of prechilled cell lysate to each well. The cell lysate was collected in a 1.5 mL centrifuge tube and centrifuged at 12000 rpm for 15 min. The supernatant was collected for protein quantification using BCA protein quantification kit (Beyotime, China). After quantifying the protein, add the loading buffer according to the ratio, prepare a loading solution with a final protein concentration of 1 *μ*g/*μ*L, and place in a 100°C water bath for 5 min to denature. After cooling at room temperature, store in a refrigerator at -20°C for later use. For SDS-PAGE gel electrophoresis, 20 *μ*g of protein was loaded on each sample at 90 V for 120 min and PVDF was transferred to the membrane at 110 V for 70 min. Tanon gel imager (Tanon, China) was used to analyze the contents of TGF-*β*1, NF-*κ*B, and TNF-*α* (all antibodies were purchased from CST, USA).

### 2.4. Establishment of Diabetic Rat Model

This study was approved by medical ethics committee of the First Hospital of Jilin University. Thirty 4-week-old male Wistar rats were kept in separate cages at a temperature of 22 ± 2°C and relative humidity of 55% ± 2%, with *ad libitum* access to food and water. Ten rats were included in the healthy control group (supplied with normal water and diet), the remaining 20 rats were fed high-fat and high-sugar diets (66.6% base material, 20% sugar, 10% lard, 0.4% cholesterol, and 3% egg yolk powder). After feeding for 4 weeks, the rats were given an intraperitoneal injection of STZ (0.1 mmol/L sodium citrate buffer, pH 4.4, 35 mg/kg body weight) once, followed by a high-fat and high-sugar diet for 1 week. Fasting blood glucose (FBG) was measured from tail blood for three days consecutively. It was considered a successful model with FBG above 16.7 mmol/L continuously. Eighteen rats that conformed to the standards of diabetes were used for the following experiments.

### 2.5. Experimental Grouping, 20(S)-Rg3 Intervention, and Serological Detection

**Figure 1:**
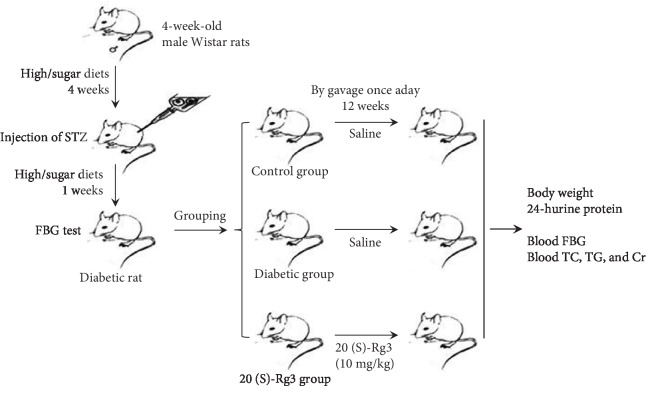
The experimental design and animal experiment flow chart. Four-week-old male Wistar rats were fed high-fat and high-sugar diets. After feeding for 4 weeks, the rats were given an intraperitoneal injection of STZ (0.1 mmol/L sodium citrate buffer, pH 4.4, 35 mg/kg body weight) once, followed by a high-fat and high-sugar diet for 1 week for diabetic model. FBG above 16.7 mmol/L continuously was considered successful modeling. The rats were randomly assigned to three groups after establishment of the diabetic model. Give saline or 20(S)-Rg3 intervention for 12 weeks. Body weight and other indicators were tested.

The rats were randomly assigned to three groups after establishment of the diabetic model (Figure 1): control group: 10 normal adult rats with 1 mL of saline by gavage once a day; diabetic group: 9 modeled diabetic rats with 1 mL of saline by gavage once a day; and 20(S)-Rg3 treatment group: 9 modeled diabetic rats were included in the treatment group with 20(S)-Rg3 (10 mg/kg body weight/day) [20] by gavage once a day. All rats were kept in plastic-bottomed cages with a controlled temperature (about 25°C) and humidity (about 60%), and a 12 h light/12 h dark cycle, and were given free access to laboratory pellet chow and water. After 12 weeks, body weights were measured. The rats were kept in metabolic cages to collect 24 h urine samples to measure specific gravity and protein content in the urine. BCA method was used to measure the urine parameters. After 12 h fasting, the rats were sacrificed with intraperitoneal injection of 2 mL 3% pentobarbital sodium. Blood was collected from the abdominal aorta to measure FBG, TC, TG, and Cr. The kidney was collected and kept in 10% neutral formalin buffer for histological examination.

### 2.6. Periodic Acid-Schiff (PAS) Staining, Immunohistochemical (IHC) Staining, and TUNEL Staining of Kidney Tissues

The kidneys were cut longitudinally into 3 mm thick pieces. The tissues were then dehydrated, transparentized, impregnated, and embedded. The well-embedded wax block was cut into paraffin sections of 3 *μ*m thickness. PAS staining, immunohistochemical staining, and TUNEL staining were performed according to the conventional methods [21, 22]. Phosphate-buffered saline (PBS) was used instead of the primary antibody as a negative control because the effective concentration of the primary antibody was 1:300. Brown particles in the nucleus or cytoplasm and higher color intensity than background staining indicated positive staining. We randomly selected eight high magnification visions of each section. The images of these high magnification visions were analyzed by IPP 6.0 software. Meanwhile, the mean integral optical density (IOD) values were tested and analyzed statistically.

### 2.7. Statistical Analysis

SPSS17.0 statistical software was used for data analysis. All data were tested for normality, and the results were expressed as $\text{means}\pm \text{standard deviations }\left(\bar{x}\pm \text{S}\right)$. Comparisons were performed by one-way ANOVA for the different groups, followed by post hoc pairwise repetitive comparisons with Turkey test. Statistical significance was set at *P* < 0.05 for all analyses.

## 3. Results

### 3.1. General Features of Experimental Animals

Of the 20 rats, 18 rats conformed to the standard of diabetes, and the success rate of establishment of the model was 90%. Seven of 9 rats in the diabetic group were alive after treatment, while 2 rats died during the 12-week period. No rats died in the control group and 20(S)-Rg3 treatment group.

The body weight, urine protein, FBG, TC, TG, and Cr content after 20(S)-Rg3 treatment for 12 weeks are presented in Table 1. The rats in the diabetic group showed significantly reduced body weight compared to the rats in the normal control group (*P* < 0.01). The 20(S)-Rg3 treatment group showed higher body weight than the diabetic group (*P* = 1.53*e* − 006). The urine protein content in the diabetic group was significantly higher than that in the normal control group (*P* < 0.01). Urine protein content in the 20(S)-Rg3 treatment group was significantly lower than that in the diabetic group (*P* < 0.01). The rats in the diabetic and 20(S)-Rg3 treatment groups had higher levels of FBG (*P* < 0.01), TC (*P* < 0.01), TG (*P* < 0.01), and Cr (*P* < 0.01) than those in the normal control group. However, 20(S)-Rg3-treated rats showed a decreasing trend of FBG, TC, TG, and Cr when compared to diabetic rats. 20(S)-Rg3 treatment improves the clinical indicators of diabetic rats.

### 3.2. 20(S)-Rg3 Prevents Diabetes-Induced Histopathological Changes in Diabetic Rats

**Figure 2:**
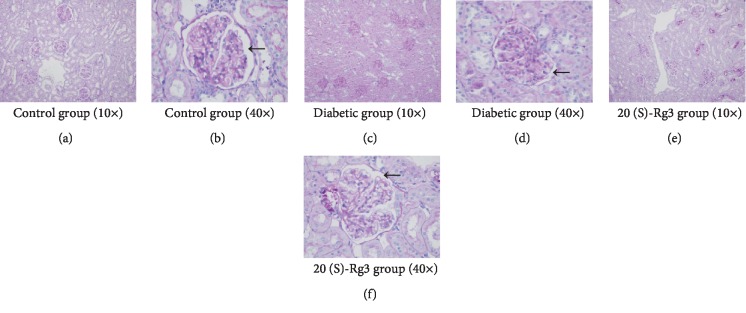
20(S)-Rg3 prevents diabetes-induced histopathological changes in diabetic rats. (a, b) PAS staining of kidney tissues in the control group. (a) and (b) are 10 times and 40 times light microscopy pictures, respectively. (c, d) PAS staining of kidney tissues in the diabetic group. (c) and (d) are 10 times and 40 times light microscopy pictures, respectively. (e, f) PAS staining of kidney tissues in 20(S)-Rg3 group. (e) and (f) are 10 times and 40 times light microscopy pictures, respectively. Black arrows point to the glomerular capillary loops.

To further detect the protective effect of 20(S)-Rg3 on DKD, we need to know the histopathological changes of the kidney after 20(S)-Rg3 intervention. PAS staining of kidney tissues in the control group showed that the glomerular capillary loops opened well, with no mesangial proliferation and thickening of basement membrane (Figures 2(a) and 2(b)). But, in the diabetic group, it showed opened glomerular capillary loops, increased glomerular volume, narrow capsule space, and mild and diffuse increase in glomerular mesangial matrix and mesangial cell hyperplasia, although clear thickening of basement membrane was not observed (Figures 2(c) and 2(d)). However, all these kidney abnormal changes were significantly prevented by the treatment with 20(S)-Rg3. After the 20(S)-Rg3 treatment, glomerular capillary loops opened well, without an obvious increase in volume. There was no clear renal capsule stenosis. Segmental and mild glomerular mesangial matrix and mesangial cell hyperplasia were observed, while the thickening of basement membrane was not significant (Figures 2(e) and 2(f)). Treatment of 20(S)-Rg3 significantly decreased the histopathological damage in diabetic rats.

### 3.3. 20(S)-Rg3 Decreases Diabetes-Induced Apoptosis in Diabetic Rats

**Figure 3:**
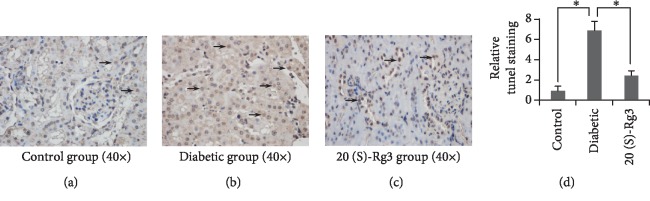
20(S)-Rg3 decreases diabetes-induced apoptosis in diabetic rats. (a–c) TUNEL staining of the kidney in different groups. Black arrows point to positive stained cell nuclei. (d) Relative qualification of the TUNEL staining of kidney in different groups. All data shown are mean values ± SD (error bar) from six replicates. ^∗^*P* < 0.01.

To define whether20(S)-Rg3 protects against diabetes-induced renal damage in diabetic rats is resulted from inhibiting the renal tubular epithelial cell apoptosis, TUNEL staining was employed. TUNEL-positive cells were mainly presented in the renal tubular epithelial cells of rats in each group (Figure 3(a)–3(c)). As what we can see (Figure 3(d)), although the rate of 20(S)-Rg3 renal tubular epithelial cell apoptosis in the diabetic and 20(S)-Rg3 treatment groups was significantly higher than that in the control group (*P* < 0.01), the apoptosis rate in the 20(S)-Rg3 treatment group was markedly lower than that in the diabetic group (*P* < 0.01). 20(S)-Rg3 treatment decreases diabetes-induced apoptosis of the renal tubular epithelial cells obviously.

### 3.4. 20(S)-Rg3 DownRegulates the Expression of Inflammatory Factors

**Figure 4:**
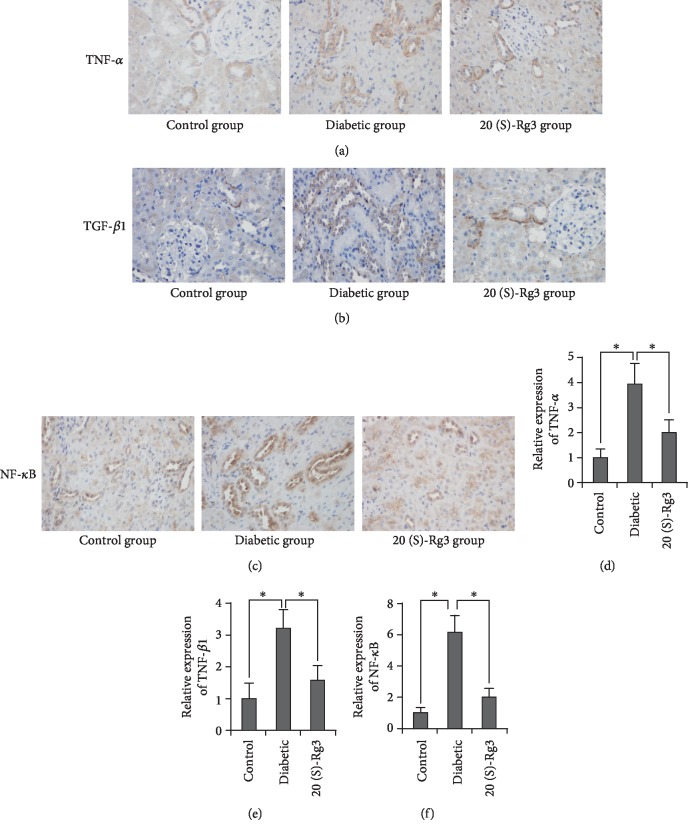
20(S)-Rg3 downregulates the expression of inflammatory factors. (a–c) IHC staining of (a) TNF-*α*, (b) TGF-*β*1, and (c) NF-*κ*Bp65 in the kidney in different groups. (d–f) Relative qualification of the IHC staining of (d) TNF-*α*, (e) TGF-*β*1, and (f) NF-*κ*Bp65 in the kidney in different groups. All data shown are mean values ± SD (error bar) from six replicates. ^∗^*P* < 0.01.

From clinical indicators to histopathological changes, the results above confirmed that 20(S)-Rg3 prevented the development of DKD in diabetic rats. Mechanistically, inflammation is crucial in the initiation and development of DKD. So, we sought to establish whether 20(S)-Rg3 could protect the kidney from diabetes by inhibiting the inflammation. According to the results of IHC staining (Figures 4(a)–4(c)), the kidneys in all groups were found to show TNF-*α*-, TGF-*β*1-, and NF-*κ*Bp65-positive staining. TNF-*α* and TGF-*β*1were expressed in the cytoplasm of renal tubular epithelial cells. NF-*κ*Bp65 were mainly expressed in the cytoplasm and nucleus of renal tubular epithelial cells when observed under an emission microscope. The IPP software was employed to analyze the positive expression intensity of three factors (Figures 4(d)–4(f)). The expression of three factors were significantly higher in the diabetic group compared with the control group (*P* < 0.01). But, after the intervention of 20(S)-Rg3 in diabetic rats, all these three inflammatory factors decreased dramatically (*P* < 0.01). These data suggest that 20(S)-Rg3 downregulates the expression of inflammatory factors, leading to significant prevention of diabetes-induced inflammation.

### 3.5. 20(S)-Rg3 Protects the NRK-52E Cells in Condition of Palmitic Acid

**Figure 5:**
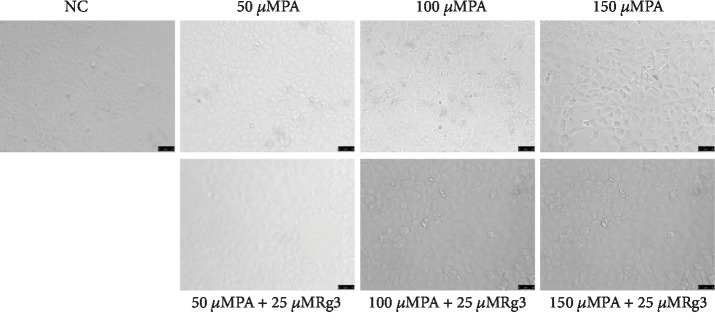
20(S)-Rg3 could decrease the round cells caused by palmitic acid. Representative images of cell expansion after treated with different concentration of PA with or without different concentration of 20(S)-Rg3 for 48 h (20x).

**Figure 6:**
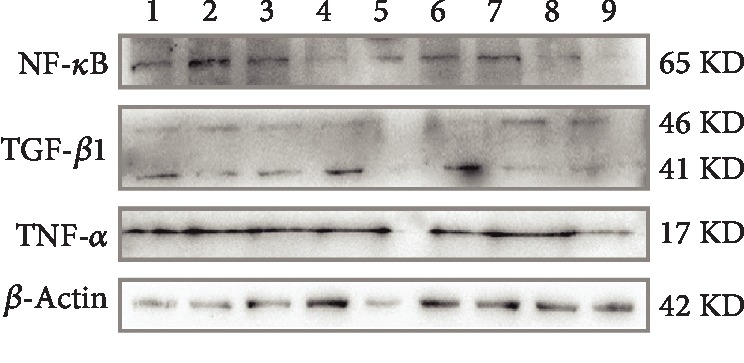
Expression of inflammatory factors in NRK-52K cells examined by western blotting. 1: 50 *μ*M Rg3; 2: 50 *μ*MPA+50 *μ*M Rg3; 3: 100 *μ*MPA+50 *μ*M Rg3; 4: 150 *μ*MPA+50 *μ*M Rg3; 5: 25 *μ*M Rg3; 6: 50 *μ*MPA+25 *μ*M Rg3; 7: 100 *μ*MPA+25 *μ*M Rg3; 8: 150 *μ*MPA+25 *μ*M Rg3; 9: NC (DMEM).

In order to further verify the beneficial effects of 20(S)-Rg3 in vitro experiments, we examined the expression of inflammatory factors and apoptosis-related factors in NRK-52E cells (renal tubular epithelial cells of rats) by western blotting. First, we treated NRK-52E cells with a different concentration of palmitic acid for 24 h to build insulin resistance cell model. It was found that NRK-52E cells became round and the cell count decreased dramatically after being treated with palmitic acid for 24 h, which showed dose dependence (Figure 5). When cells were added with 25 *μ*M 20(S)-Rg3, the round cells decreased (Figure 5). Then, we examined the expression of inflammatory factors in NRK-52E cells treated with 100 *μ*M PA with or without 20(S)-Rg3 by western blotting. We found that the expression of NK-*κ*B, TGF-*β*1, and TNF-*α* increased when cells were treated with palmitic acid. But the expression of these factors did not decreased after intervention of 20(S)-Rg3 obviously (Figure 6).

## 4. Discussion

This study indicated, for the first time, that treatment with 20(S)-Rg3 could significantly protect the kidney from the pathological process of DKD in the diabetic model, and 20(S)-Rg3 also significantly downregulated the expression of inflammatory factors, leading to significant prevention of diabetes-induced inflammation.

20(S)-Rg3 is a compound that has many biological effects of preventing ischemia-reperfusion injury, reducing oxygen free radicals, antioxidation, and inhibiting the activity of voltage-regulated pathways. Studies have reported that 20(S)-Rg3 as a nontraditional therapy has protective effects on diabetes itself, as well as diabetic cardiomyopathy, diabetic retinopathy, and atherosclerosis in DM [23, 24]. Kang et al. [20] reported 20(S)-Rg3 prevents the progression of renal damage and dysfunction in type 2 diabetic rats via inhibiting oxidative stress and advanced glycation end-product formation. In the present study, we also found the protective effects of 20(S)-Rg3 on DKD. However, these effects were achieved by down-regulating the expression of inflammatory factors, leading to significant prevention of diabetes-induced inflammation.

DKD is one of the most serious complications of diabetes, and the most important pathogenesis of DKD is inflammation. In the early stages of DKD, the increase in glucose level changes the renal hemodynamics, and consequently, the pressure in the glomerulus increases, thereby increasing the filtration rate. When this state lasts, it can damage endothelial cells, thicken the basement membrane, and thus form the pathologic basis of DKD. Inflammation caused by hyperglycemia and lipid metabolism disorder leads to the releasing of a variety of inflammatory factors in combination with other mechanisms to promote the inflammatory reactions, accelerates the above process and development of DKD [25]. So, inhibition of inflammation is an important target for a treatment of DKD. After the intervention of 20(S)-Rg3 in diabetic rats, all the three inflammatory factors, TGF-*β*1, NF-*κ*Bp65, and TNF-*α*, decreased dramatically. Although the expression of these factors did not decrease after intervention of 20(S)-Rg3 in western blot, it may be the effect of DMSO used to dissolve the 20(S)-Rg3 powder (concentration of 2.5% DMSO in cell medium). More experiments should be performed to modify the protection of 20(S)-Rg3 by western blotting. The anti-inflammation effect of 20(S)-Rg3 may, furthermore, decrease diabetes-induced apoptosis of the renal tubular epithelial cells, and the kidney histopathological damage in the end. There was no clear renal capsule stenosis. In vitro experiment, we found that 20(S)-Rg3 could decrease the round cells caused by palmitic acid. Segmental and mild glomerular mesangial matrix and mesangial cell hyperplasia were observed, while the thickening of basement membrane was not significant, and glomerular capillary loops opened well, without an obvious increase in volume.

The improvement of the clinical indicators in diabetic rats further confirmed the protective effect of 20(S)-Rg3 on DKD. Diabetic rats treated with 20(S)-Rg3 weight regained and urine protein decreased significantly; glucose and lipid metabolism indicators also showed down tendency compared to DKD rats. Conversely, weight regained and improvement in metabolic indicators will further help control the development of DKD. Because high blood glucose fluctuations may damage glomerular mesangial cells and promote cell apoptosis, and lipid metabolism disorder and abnormal lipid deposition in the glomerulus may lead to proliferation of glomerular cell membrane, causing gradual accumulation of ECM, which explains the pathological basis of DKD [26].

We are also aware of a few limitations in the present study. We used only one concentration of 20(S)-Rg3 treatment although the effect of 20(S)-Rg3 showed dose-dependence in the previous study [27]. But the current dose of 20(S)-Rg3 is based on a report of the recent study with the most dramatic protective effects [20] and the preexperiment with different concentrations of 20(S)-Rg3 in [Supplementary-material supplementary-material-1] in the supplemental materials. And the other more inflammatory factors and apoptosis-related factors could be detected in order to further confirm our finding.

In summary, our study identified the novel mechanism and efficacy of 20(S)-Rg3 as a nontraditional therapy on protection of renal damage in diabetic rats. Plus, there are also the beneficial effects of 20(S)-Rg3 on hyperglycemia, hyperlipidemia, and body weight. 20(S)-Rg3 has great potential for use in the clinical realm for the prevention and treatment of DKD.

## Figures and Tables

**Table 1:** General indicators of rats in each group.

	Control group (*n* = 10)	Diabetic group (*n* = 7)	20(S)-Rg3 treatment group (*n* = 9)
Body weight (g)	384.86 ± 18.69	276.86 ± 13.23^∗^	363.57 ± 22.71^#^
Urine protein (mg/24 h)	1.28 ± 0.44	4.42 ± 1.48^∗^	2.28 ± 0.90^#^
FBG (mmol/L)	5.87 ± 0.33	24.53 ± 6.30^∗^	21.87 ± 5.59
Cr (mmol/L)	85.71 ± 9.96	114.57 ± 10.37^∗^	107.14 ± 14.46
TC (mmol/L)	0.45 ± 0.07	6.43 ± 1.15^∗^	5.59 ± 0.97
TG (mmol/L)	1.15 ± 0.05	4.74 ± 1.12^∗^	4.01 ± 0.49

^∗^ *P* < 0.001 compared with the control group, ^#^*P* < 0.01 compared with the diabetic group.

## Data Availability

The table and figure data used to support the findings of this study are included within the article.

## Abbreviations

20(S)-Rg3: 20(S)-Ginsenoside Rg3
20(R)-Rg3: 20(R)-Ginsenoside Rg3
CAT: Catalase
Cr: Creatinine
CTGF: Connective tissue growth factor
DAB: Diaminobenzidine
DKD: Diabetic kidney disease
DM: Diabetes mellitus
ECM: Extracellular matrix
FBG: Fasting blood glucose
GSH: Glutathione- SH
IOD: Integral optical density
MDA: Malonaldehyde
NF-*κ*B: Nuclear transcription factors-kappa B
PAS: Periodic acid-Schiff
PDGF: Platelet-derived growth factor
SOD: Superoxide dismutase
SP: Surfactant protein
STZ: Streptozotocin
TC: Total cholesterol
TG: Triglycerides
TGF-*β*1: Transforming growth factor beta 1
TNF-*α*: Tumor necrosis factor-*α*
VEFG: Vascular endothelial cell growth factor.

## Supplementary Materials

Supplementary Materials The clinical characteristics of all the rats in each group in the preexperiments.
