# Supplementary material for: 20(S)-Ginsenoside Rg3 Protects Kidney from Diabetic Kidney Disease via Renal Inflammation Depression in Diabetic Rats
Source: J Diabetes Res. 2020 Mar 18;2020:7152176. doi: 10.1155/2020/7152176 (PMC7106937; doi:10.1155/2020/7152176)
Supplement: Supplementary Materials — The clinical characteristics of all the rats in each group in the preexperiments. [file 7152176.f1.docx]

Table S1 General indicators of rats in each group in pre-experiments ( $\bar{\text{x}}$±S)

| grups | body weight (g) | FBG(mmol/L) | TC(mmol/L) | TG(mmol/L) |
| --- | --- | --- | --- | --- |
| control | 343.40±30.25 | 5.88±0.86 | 0.448±0.08 | 1.15±0.13 |
| diabetic | 253.20±9.98 | 24.34±10.73 ^a^ | 6.47±1.55 ^a^ | 4.65±1.38 ^a a^ |
| 5.0mg/kgRg3 | 261.60±14.8 | 24.3±9.42 | 6.45±1.35 | 4.40±2.11 |
| 10.0mg/kgRg3 | 274.20±13.37 | 23.8±7.19 | 6.11±1.96 | 4.11±3.24 |
| 20.0mg/kgRg3 | 296.60±23.43 | 21.68±11.56 | 5.46±0.83 | 3.72±0.20 |
